# Simply Prepared Magnesium Vanadium Oxides as Cathode Materials for Rechargeable Aqueous Magnesium Ion Batteries

**DOI:** 10.3390/nano12162767

**Published:** 2022-08-12

**Authors:** Milica M. Vasić, Miloš Milović, Danica Bajuk-Bogdanović, Tamara Petrović, Milica J. Vujković

**Affiliations:** 1 Faculty of Physical Chemistry, University of Belgrade, Studentski trg 12-16, 11000 Belgrade, Serbia; 2 Institute of Technical Sciences of SASA, Knez Mihajlova 35/IV, 11000 Belgrade, Serbia

**Keywords:** magnesium vanadium oxides, rechargeable magnesium batteries, aqueous electrolyte, cyclic voltammetry, charge storage improvement

## Abstract

Vanadium-oxide-based materials exist with various vanadium oxidation states having rich chemistry and ability to form layered structures. These properties make them suitable for different applications, including energy conversion and storage. Magnesium vanadium oxide materials obtained using simple preparation route were studied as potential cathodes for rechargeable aqueous magnesium ion batteries. Structural characterization of the synthesized materials was performed using XRD and vibrational spectroscopy techniques (FTIR and Raman spectroscopy). Electrochemical behavior of the materials, observed by cyclic voltammetry, was further explained by BVS calculations. Sluggish Mg^2+^ ion kinetics in MgV_2_O_6_ was shown as a result of poor electronic and ionic wiring. Complex redox behavior of the studied materials is dependent on phase composition and metal ion inserted/deinserted into/from the material. Among the studied magnesium vanadium oxides, the multiphase oxide systems exhibited better Mg^2+^ insertion/deinsertion performances than the single-phase ones. Carbon addition was found to be an effective dual strategy for enhancing the charge storage behavior of MgV_2_O_6_.

## 1. Introduction

Today, lithium-ion batteries are used in numerous electronic devices and electric vehicles because of their stable cycle life, light weight, and high capacity [1,2]. In spite of this, limited lithium resources, relatively high costs, and operational safety problems bring their future massive application into question [3,4,5]. Therefore, considerable research efforts have been devoted towards development of alternative battery systems [5,6,7,8,9,10,11,12], based on Earth’s abundant elements such as Na [6,13], Mg [5], Al [7,10,14], Zn [8], etc. Challenging concepts such as solid-state [13] and aqueous rechargeable battery systems [15,16] have been developed to achieve safe and sustainable energy storage technology. Rechargeable magnesium (RMBs), sodium (RSBs), and aluminum (RABs) are considered very promising candidates for partial replacement of the lithium-ion batteries, due to high natural abundance of elements (Mg, Na, and Al) and resulting lower costs of these batteries than the Li ones, and their better atmospheric stability, safety of handling, and eco-friendliness [5,9,17,18]. Regarding Na and Al storage systems, critical issues have been successfully addressed [13,14] with the aim to design materials and interfaces for their practical application.

The choice of host material for the storage of Mg^2+^ is of huge importance for the development of RMBs. Layered intercalation materials offer advantages of relatively high capacity and good structural stability, while the ion intercalation is allowed by their large interlayer space [5,19,20]. Vanadium oxides have been attracting attention as layered materials potentially applicable for RMBs since the first publication about the ability of V_2_O_5_ to reversibly electrochemically intercalate Mg^2+^ at room temperature [17]. Transportation of Mg^2+^ ions in α-V_2_O_5_ phase is often slow because of strong interactions between Mg^2+^ and neighboring O atoms [5,21]. However, defect engineering (ionic vacancy, doping, amorphous phases, and phase interfaces) and solvation effects may facilitate diffusion of bivalent ions [22,23]. Furthermore, density functional theory investigation revealed that, by increasing magnesium intercalation concentration, α-phase V_2_O_5_ host material can be converted into ε and δ phase [24]. So far, different vanadium-oxide-based materials have been synthesized and tested as possible cathodes for RMBs [5,18,22,25,26,27,28,29]. Various preparation methods have been used for that purpose, including hydrothermal or solvothermal synthesis, usage of microwave irradiation, conventional heat-treatment, sol–gel method, etc. [30,31,32,33,34,35].

The advantages of the use of aqueous electrolytes over nonaqueous ones in the first place include lower toxicity, flammability, environmental risks, and costs [8,36,37], while improved Mg^2+^ kinetics is also expected in oxide cathodes [17,18,38,39]. In general, the kinetics of multivalent ions is more difficult than the kinetics of alkaline ions due to the stronger interaction of multivalent ions with structural and electrolyte surrounding. On the other hand, double and triple charge can provide higher charge transfer, while the co-intercalated water can protect multivalent ions from strong interaction with the surrounding environment [40], thus making structure more stable during cycling. Recently, great success has been achieved with Zn and Al aqueous electrochemistry [15,16], while similar achievements seem to be still elusive for Ca and Mg aqueous chemistry. Nevertheless, Mg-ion aqueous batteries may provide certain advantages over Zn and Al aqueous systems, in terms of the cyclic stability. For example, better cyclic stability of the sodium vanadate cathode has been demonstrated in Mg- than in Al-containing aqueous electrolytes [41], as a result of weaker interaction of bare Mg^2+^ than Al^3+^ ion with the structure and different pH of the electrolyte. Further, Mg-ion aqueous chemistry does not suffer from dendrite issues as is the case with Zn-aqueous ion batteries. 

A number of studies have been published on rechargeable aqueous batteries containing vanadium-oxide-based cathode materials [42,43,44,45,46,47,48,49,50], pointing out the importance of appropriate choice of electrode structure for battery performances. In this work, several Mg-V-O materials synthesized by simple preparation route were studied and discussed as potential cathodes for aqueous RMBs. Addition of carbon to the Mg-V-O was considered as a strategy for the charge storage improvement.

## 2. Experimental Part

For the synthesis of the MgVO-HT material (composition MgV_2_O_6_), MgVO precursor was prepared by precipitation method [51,52], using NH_4_VO_3_ and Mg(OH)_2_ reactants mixed at the Mg:V molar ratio 1:2. Namely, NH_4_VO_3_ was dissolved in 1 wt.% NH_4_OH solution to make 0.5 M NH_4_VO_3_, which was subsequently mixed with the appropriate amount of Mg(OH)_2_. The mixture was evaporated to dryness with stirring, and then dried at 100 °C. The obtained precursor was ground in an agate mortar, and finally calcined in air at 550 °C (sample denoted as MgVO-LT) or successively at 550 and 700 °C (denoted as MgVO-HT), for 12 h at each temperature.

MgVO materials of the compositions Mg_2_V_2_O_7_ and Mg_3_V_2_O_8_, prepared for comparison purposes, were made in a manner analogous to that of the MgVO-HT, but with the reactants ratio appropriate to the desired compositions, and final calcination temperature of 800 °C in the case of Mg_3_V_2_O_8_. 

To obtain MgVO material with improved electrochemical properties, 100 mg of MgVO-HT material was mixed with 31 mg of sucrose, and the mixture was thermally treated at 700 °C for 2 h under Ar atmosphere. The material prepared in this way was named MgVO/C.

X-ray diffraction measurements (XRD) were performed using Philips PW 1050 X-ray powder diffractometer, in Bragg–Brentano focusing geometry, with Ni-filtered Cu Kα radiation. For the analysis of the XRD patterns, ICSD database [53] was used. Crystallite size of MgV_2_O_6_ phase was estimated applying Scherrer equation [54] to the peak positioned at 14.3° (2θ). 

Scanning Electron Microscopy (SEM) measurements were performed by means of the SEM device Phenom ProX. 

Fourier transform infrared spectra (FTIR) were collected using a Thermo Scientific IS20 Nicolet device, with the KBr technique. The measurements were carried out with 32 scans per spectrum and 4 cm^−1^-resolution. Raman spectroscopy was performed with a Thermo Scientific DXR Raman microscope, using an excitation wavelength of 532 nm and power of 8 mW. The measurements included 10 exposures per spectrum, 10 s exposure time, and pinhole size of 50 μm. The objective magnification was 10×. For the UV–Vis spectroscopic measurements, Thermo Scientific Evolution 220 UV-Visible Spectrophotometer was employed.

Electrochemical measurements were carried out in a three-electrode cell, with a Gamry PCI4/300 potentiostat/galvanostat. Saturated calomel electrode (SCE) was used as a reference electrode, and a platinum foil as a counter electrode. Working electrodes were prepared by depositing a paste consisting of active material, Vulcan carbon black, and poly-vinylidenefluoride (PVDF) (2 wt.% PVDF in N-methyl-2-pyrrolidone) at the weight ratio 85:10:5 onto the glassy carbon rods, and drying the rods under vacuum overnight. All the measurements were performed in aqueous solutions of nitrate salts (Mg, Al, or Li) at room temperature. The values of specific current presented in the paper are given relative to the weight of active material.

For the insight of possible Mg^2+^ diffusion pathways in the structures of interest, the bond valence method has been utilized. Bond valence sum (BVS) calculations were carried out in 3DBVSMAPPER [55] software. The method is based on the valence sum rule and includes calculation of bond valence sum value for the chosen ion (in this case, Mg^2+^) located at the particular point in the crystal by measuring distances to the neighboring counter-ions (in this case, O^2−^) [56]. Assuming that any point in the unit cell with the BVS value for Mg^2+^ close to 2 represents a possible location of Mg^2+^ to appear there, the BVS distribution on a 3D grid reveals possible locations of Mg^2+^ occurrence in the lattice. By obtaining volume for a given BVS mismatch (in our case, 2 ± 0.1 valence units), the method is readily used for visualizing a 3D map of diffusion pathways of some particular ion [55].

## 3. Results and Discussion

### 3.1. Structural Characterization

**Figure 1:**
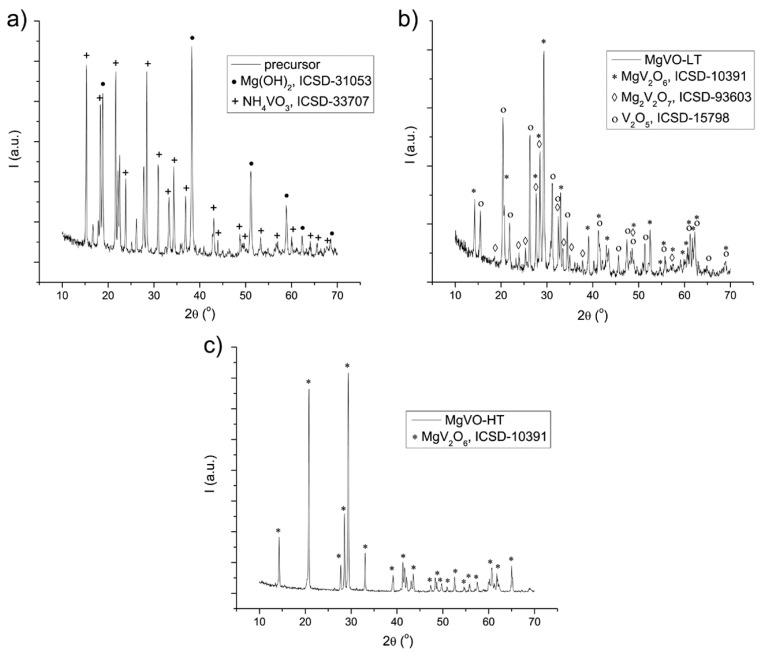
XRD patterns of the MgVO precursor (**a**); MgVO-LT (**b**); and MgVO-HT (**c**) material.

Structural characterization of the MgVO precursor was performed before and after thermal treatments by means of XRD and vibrational spectroscopy techniques (FTIR and Raman spectroscopy). According to the XRD results, the nonthermally treated precursor is a mixture composed of Mg(OH)_2_ and NH_4_VO_3_ phases (Figure 1a). After 12 h annealing at 550 °C in air, the precursor phases were transformed into the composite material containing V_2_O_5_, MgV_2_O_6_, and Mg_2_V_2_O_7_ phases with the weight fractions 31%, 51%, and 18%, respectively, designated as MgVO-LT (Figure 1b). Finally, after further annealing of the MgVO-LT at 700 °C for 12 h in air, the single-phase material consisting of MgV_2_O_6_ phase is obtained (Figure 1c) and named MgVO-HT.

**Figure 2:**
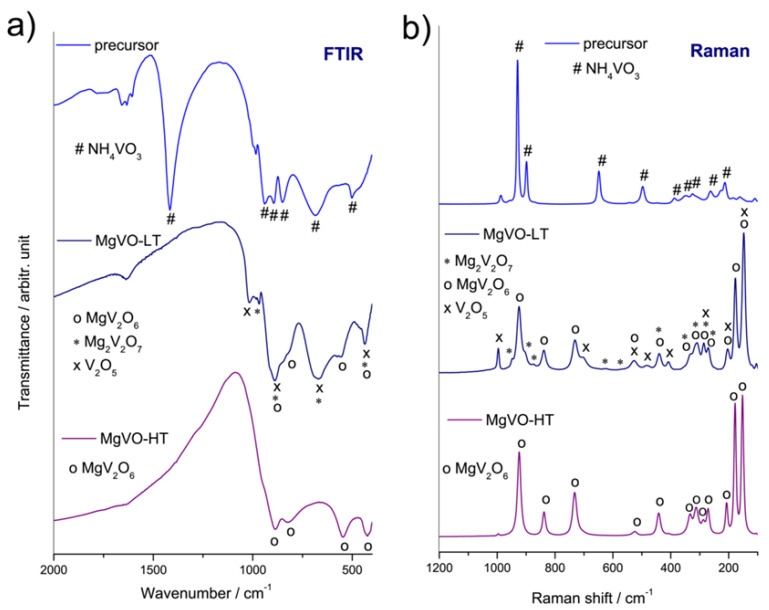
FTIR (**a**) and Raman (**b**) spectra of the MgVO precursor, MgVO-LT, and MgVO-HT material.

Vibrational spectroscopy analysis yielded results consistent with those obtained using the XRD analysis. The vibrational band of water, i.e., humidity of the samples, is observed at around 1635 cm^−1^ in the FTIR spectra of all the samples, resulting from O-H-O scissors-bending vibrations [57]. Characteristic modes of MgV_2_O_6_ phase [58,59,60,61,62,63] can be seen in the FTIR and Raman spectra of both MgVO-LT and MgVO-HT samples (Figure 2). Besides those of MgV_2_O_6_, vibrational modes of V_2_O_5_ and Mg_2_V_2_O_7_ phases [58,59,60,61,62,63] can be recognized in the FTIR and Raman spectra of the MgVO-LT, as assigned in Figure 2 and Table 1 and Table 2. For the mentioned phases, stretching V=O vibrational modes can be found in the region 920–1030 cm^−1^, while the stretching asymmetric VO_4_ and VO_6_ ones can be seen in the wavenumber range 815–920 cm^−1^ (Figure 2). Spectral bands in the regions 620–740 cm^−1^ and 520–580 cm^−1^ can be associated with the stretching asymmetric and symmetric V-O-V vibrations, respectively. The bending symmetric VO_4_ and VO_6_ vibrational modes can be found in the region 410–450 cm^−1^. Detailed assignation of individual bands of the Mg-V-O phases is presented in Table 1 and Table 2. Vibrational modes of the NH_4_VO_3_ dominate the spectra of the precursor material, (Table S1, Supplementary Materials). In the Raman spectra, the high-frequency MgV_2_O_6_ modes (205 and 150 cm^−1^) become slightly shifted in the vanadate mixture (for a few cm^−1^ towards higher frequencies), as a result of the interaction with other phases and coupling of their modes.

### 3.2. Morphological Characterization

**Figure 3:**
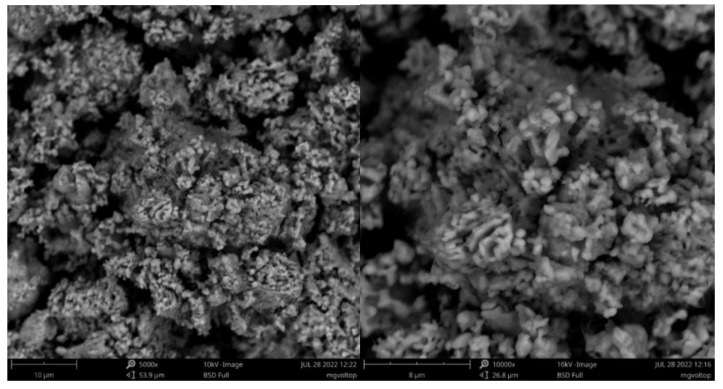

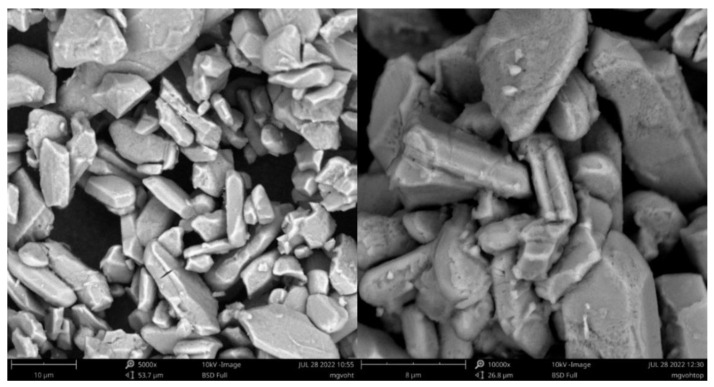
SEM micrographs of MgVO-LT (**up**) and MgVO-HT (**down**).

SEM measurements of MgVO-LT and MgVO-HT samples (Figure 3) clearly confirm their morphological difference, which occurs as a result of the applied annealing temperature and the formed phase composition. At first glance, MgVO-LT microparticles (10–20 μm) are composed of irregular loose nanospherical particles (Figure 3, up). Still, a certain part of them remained undecorated (visibly better in Figure S1 (left)). The observed nanoparticles’ looseness is favorable for easy diffusion of electrolyte ions. If we look closely, there are also some nanowires in the sample, visibly better at larger magnifications (Figure S1, right). It seems there are more nanowires in the bulk; so, they need more time to be activated during cycling. Three types of MgVO-LT particles can be correlated with the appearance of three phases in MgO-LT sample, as identified by XRD. The formation of a pure MgV_2_O_6_ phase upon increasing temperature completely changes the morphology of the sample. Large microagglomerates (5–20 μm) without a defined nanostructure dominate the morphology of MgVO-LT sample.

Based on the morphological observations, the polyphase material (V_2_O_5_/MgV_2_O_6_/Mg_2_V_2_O_7_) is expected to show better diffusion behavior than single-phase MgV_2_O_6_ material, which will be investigated in the following sections.

### 3.3. Electrochemical Characterization

#### 3.3.1. MgVO-HT Material

**Figure 4:**
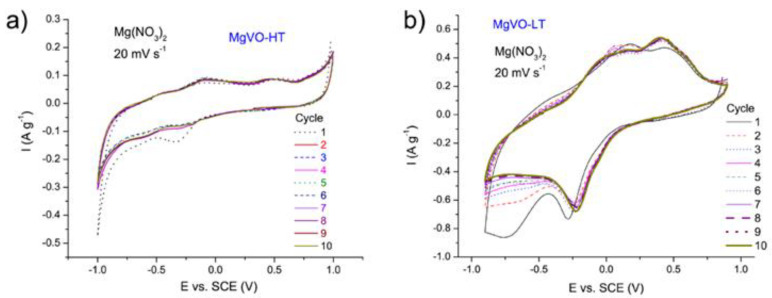

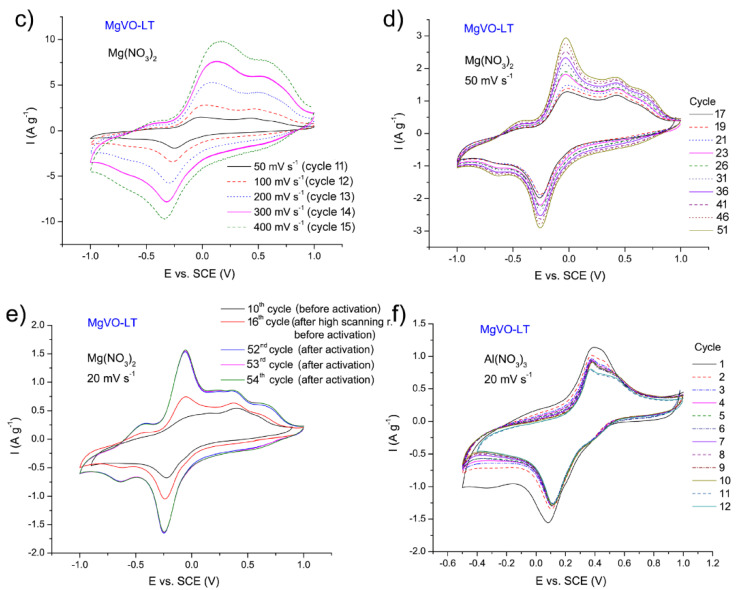
Cyclic voltammograms of MgVO-HT in 3M Mg(NO_3_)_2_ at 20 mV s^−1^ (**a**); MgVO-LT in 3M Mg(NO_3_)_2_ at 20 mV s^−1^ (**b**) and at higher scanning rates (**c**) before activation; activation of the MgVO-LT in 3M Mg(NO_3_)_2_ at 50 mV s^−1^ (**d**); MgVO-LT in 3M Mg(NO_3_)_2_ at 20 mV s^−1^ before and after activation (**e**); MgVO-LT in 1M Al(NO_3_)_3_ at 20 mV s^−1^ (**f**).

Since the simply prepared MgVO-HT is a single-phase material, its electrochemical properties are discussed first, followed by an examination of the MgVO-LT material. Cyclovoltammetric (CV) characterization of the MgVO-HT material in 3M Mg(NO_3_)_2_ solution revealed three poorly defined redox pairs (Figure 4a), resulting from the insertion/deinsertion of Mg^2+^ ions at different structural sites, including changes in the vanadium valence state [18]. By repeating CV measurements, current values were shown to be unstable. Quite low current values were obtained, which can be attributed to the low electrical conductivity of the sample. 

#### 3.3.2. MgVO-LT Material

The subject of further analyses was the multiphase composite MgVO-LT material. According to the electrochemical test in 3M Mg(NO_3_)_2_, performed by recording CVs at 20 mV s^−1^, the current became stabilized after 7 cycles (Figure 4b). CVs at higher scanning rates retained the shape of those at 20 mV s^−1^ (Figure 4c). An additional scan at 20 mV s^−1^, conducted in order to check the electrochemical performance after scanning at higher rates, revealed an unexpected increase in current values (Figure 4e). Then, further activation was performed by repeated cycling at 50 mV s^−1^ up to the stabilization (35 cycles) (Figure 4d), after which successive scans at 20 mV s^−1^ demonstrated stable current values (Figure 4e). The activation including growth of current values up to the stabilization is a result of water molecules entering the structure of the MgVO-LT [8]. Although not very high, maximal current values observed for the composite MgVO-LT material were more than 10 times higher than those obtained for simply prepared pure MgV_2_O_6_ phase (Figure 4a,e).

Positions of the cathodic peaks of the MgVO-LT, as well as of MgVO-HT, around −0.20 and −0.62 V vs. SCE (at 20 mV s^−1^), correspond to those published in the literature for the V_2_O_5_ xerogel/graphite composite in the saturated aqueous solution of Mg(NO_3_)_2_ [40], suggesting that, in the studied materials, the Mg^2+^ ions were electrochemically inserted at the sites equivalent to those published for the mentioned V_2_O_5_ composite [40]. This would mean that inserted Mg^2+^ ions occupied the sites in the structure near the center of square-planar oxygen atoms, as well as those around apical oxygen atoms [40,64]. In the anodic direction, the peak at around −0.43 V coincides with that of the aforementioned literature [40], but this is not the case at potentials more positive than −0.20 V, where the shape of the CV curves and peak positions differ from those from [40] (Figure 4). This suggests that the main Mg^2+^ extraction process from MgVO-LT is faster when compared to V_2_O_5_-based xerogel (the less positive main anodic peak) due to higher crystallinity of oxides in the mixture and different Mg^2+^- ion surroundings.

Additional testing of the MgVO-LT material was carried out in 1M Al(NO_3_)_3_ by recording CVs at 20 mV s^−1^ (Figure 4f). In this case, the observed redox peaks correspond to the insertion/deinsertion of Al^3+^ ions into/from the vanadium oxide mixture. Unlike in the Mg(NO_3_)_2_ solution, pronounced decline in the current values was observed with each subsequent CV cycle recorded in the Al(NO_3_)_3_ electrolyte (Figure 4f), suggesting damage of the electrode material with the electrochemical treatment. This resulted from mechanical strain in the structure growing with the progress of the insertion process [28] and was also contributed by the higher acidity of the Al(NO_3_)_3_ than the Mg(NO_3_)_2_ solution. Anyway, the maximal current values observed for the MgVO-LT material in Al(NO_3_)_3_ solution were lower than those in Mg(NO_3_)_2_, but significantly higher than the current values reached for the MgVO-HT in the Al(NO_3_)_3_ solution of the same concentration (Figure 4f and Figure S2, Supplementary Materials).

### 3.4. Characterization of the MgVO-LT Material after Electrochemical Treatment

**Figure 5:**
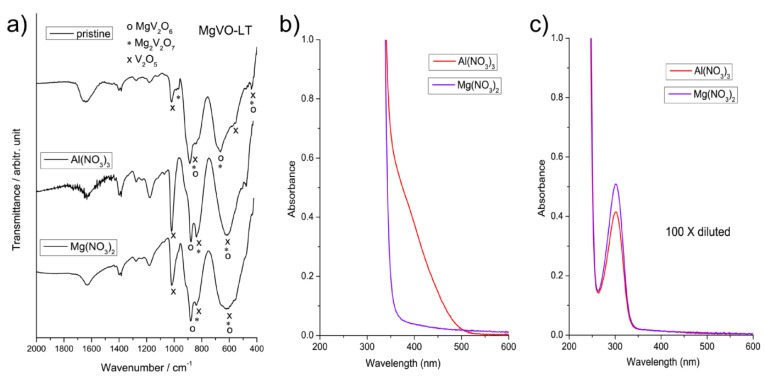
FTIR spectra of the MgVO-LT working electrodes before and after electrochemical measurements in Mg(NO_3_)_2_ and Al(NO_3_)_3_ electrolytes (**a**); UV–Vis absorption spectra of the Mg(NO_3_)_2_ and Al(NO_3_)_3_ electrolytes without dilution (**b**) and 100× diluted (**c**), after electrochemical measurements with MgVO-LT electrodes.

For a deeper insight into the behavior of the MgVO-LT material in the studied electrolytes, further examinations were performed. FTIR spectroscopy analysis of the working electrodes MgVO-LT after electrochemical measurements revealed certain changes in the vibrational bands of the electrode material, as a consequence of the electrochemical treatments in the Mg(NO_3_)_2_ and Al(NO_3_)_3_ electrolytes (Figure 5a). UV–Vis absorption spectra of the electrolytes previously used in the electrochemical measurements exhibited a peak only at around 300 nm, which corresponds to the presence of nitrate ions [65], for both 100× diluted electrolytes and undiluted Mg(NO_3_)_2_ (Figure 5b,c). However, an additional absorption band in the range 350–500 nm can be observed in the UV–Vis spectrum of undiluted Al(NO_3_)_3_ electrolyte, which can be related with the presence of dissolved vanadium (V) species in the electrolyte [66,67,68], confirming degradation of the electrode material during the electrochemical measurements in Al(NO_3_)_3_ electrolyte.

Comparison of the FTIR spectra of the electrode paste before and after electrochemical measurements showed, after the measurements, an increase in the vibrational band of V_2_O_5_ at 1020 cm^−1^ (stretching ν(V-O) [58]) relative to the vibrational bands of MgV_2_O_6_ and Mg_2_V_2_O_7_ (Figure 5a), suggesting partial degradation of the MgV_2_O_6_ and Mg_2_V_2_O_7_ phases during the electrochemical treatments in Mg(NO_3_)_2_ and Al(NO_3_)_3_. The peak at 975 cm^−1^, resulting from stretching ν(V^V^=O) and ν_s_(VO_4_) (symmetric) vibrations of Mg_2_V_2_O_7_ [58], disappeared in the spectra after the treatments, especially in the case of Al-redox process. Further, instead of merged peaks at 815–920 cm^−1^ corresponding to the vibrations of all three phases, observed in the as-prepared electrode paste, two separate peaks at around 880 and 830 cm^−1^ appeared in the spectrum after the electrochemical experiments (Figure 5a). This peaks can be ascribed to the stretching asymmetric ν_as_(VO_4_) and ν_as_(VO_6_) vibrations of the MgV_2_O_6_ and V_2_O_5_ phases [58], respectively. Most probably, the separation of the peaks resulted from weakening of the stretching asymmetric vibrational bands of the Mg_2_V_2_O_7_ phase, which were expected to appear at around 873 and 850 cm^−1^, as a consequence of partial deficiency of this phase in the electrode paste after the electrochemical treatments. These indicators of partial degradation of the electrode material were more pronounced for the Al(NO_3_)_3_ than for the Mg(NO_3_)_2_ electrolyte (Figure 5a). Structural stability of the V_2_O_5_ phase was probably improved due to insertion of the metal ions within the V_2_O_5_ structure during the electrochemical experiments [69] and introduction of hydroxyl groups at the oxide surface [41]. Broadening of the vibrational bands in the range 500–720 cm^−1^ after electrochemical measurements could be a consequence of certain structural distortions in the phases present in the electrode material, including changes in the interlayer distances [70], resulting from the Mg^2+^ or Al^3+^ ion insertion into their structures. The results of the Raman spectroscopy of the electrochemically treated samples are in agreement with the findings obtained from the FTIR analysis, (Figure S3, Supplementary Materials), but do not offer unambiguous conclusions, because of phase heterogeneity of the samples and dependence of micro-Raman spectra appearance on the sites in the samples chosen for the analysis.

#### Bond Valence Sum (BVS) Calculations for the Phases Present in the MgVO-HT and MgVO-LT

**Figure 6:**
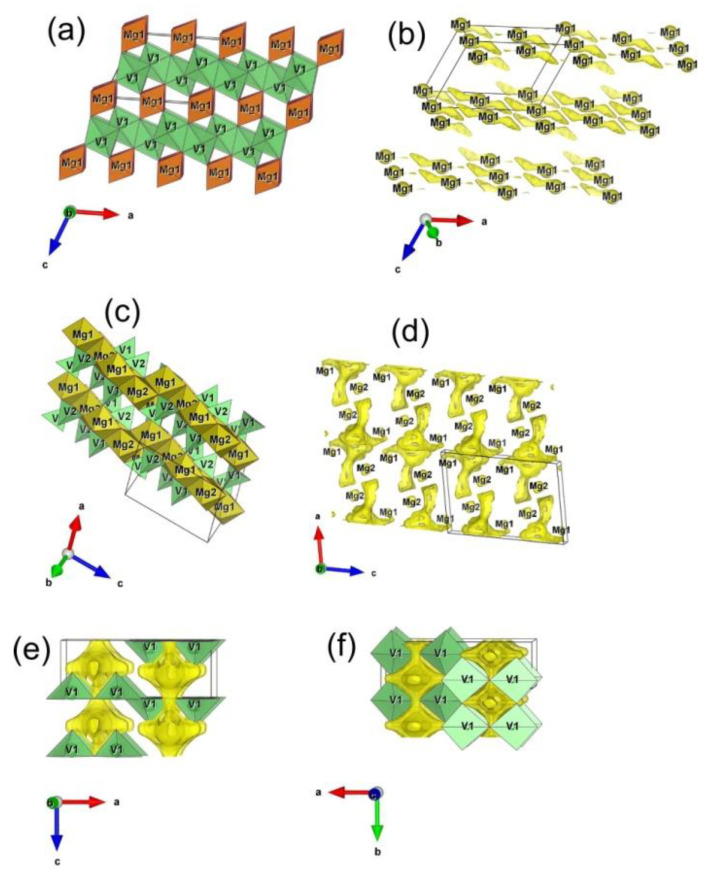
Models of migration pathways for Mg^2+^ ion obtained by BVS analysis of the phase constituents of MgVO-LT. Polyhedral representation of Mg_2_VO_6_ (**a**) and BVS map within its structure (**b**). Polyhedral representation of α-Mg_2_V_2_O_7_ (**c**) and BVS map within its structure (**d**). View on c- (**e**) and d-channels (**f**) within α-V_2_O_5_. BVS volumes are defined by ±0.1 v.u. mismatch from the valence +2 of Mg.

Let us explain redox behavior of the vanadate mixture from the aspect of the bond valence method (BVS), which helps obtain insight into possible Mg^2+^ diffusion pathways of the examined vanadium oxide structures. In our study, crystallographic data of MgV_2_O_6_ (monoclinic s. g. *C*2/*m* [71]), V_2_O_5_ (orthorhombic s. g. *Pmmn* [72]), and Mg_2_V_2_O_7_ (monoclinic s. g. *P*2_1_/*c* [73]) have been used as input for BVS calculation and its results are presented in Figure 6.

In MgV_2_O_6_, magnesium occupies 2/*m* octahedral Mg1 sites between VO_n_ layers [71], thus forming chains of edge-sharing octahedra VO_6_ along b-direction (Figure 6a). Although this seems the most obvious path for Mg^2+^ ion diffusion, there is no continuous network of BVS isosurfaces (Figure 6b). Therefore, Mg^2+^ ion migration within this structure is inherently hindered to a considerable degree, providing only low current densities, as confirmed by electrochemical measurements.

In alpha Mg_2_V_2_O_7_ structure, magnesium occupies two crystallographic positions, Mg1 and Mg2, also in octahedral coordination and forms zig-zag chains of edge-sharing Mg1O_6_ and Mg2O_6_ octahedra alternating along c-direction (Figure 6c). Both positions Mg1 and Mg2, however, remain completely isolated from the rest of the isosurface area (Figure 6d), rendering this structure more/less inactive for reversible magnesium extraction. This is in agreement with the results of cyclovoltammetric characterization of the Mg_2_V_2_O_7_ material, given in Figure S4a (Supplementary Materials).

In contrast to MgV_2_O_6_ and Mg_2_V_2_O_7_, which provide only limited capabilities for Mg^2+^ ion transfer, the alpha V_2_O_5_ structure offers two solid intercrossed channels for Mg^2+^ diffusion along the c- and b-axis, as displayed in Figure 6e,f. In this manner, a continuous network is formed for unobstructed 2D migration of Mg^2+^ within this structure. The observation is in accordance with the cyclic voltammetry measurements, which evidence substantially higher currents obtained for the MgVO-LT sample (composite of abovementioned phases) than MgVO-HT (which is plain MgV_2_O_6_). 

On the other hand, the contribution of Mg vanadate phases to the redox activity of the MgVO-LT cannot be neglected. The electrolyte remained colorless upon cycling of vanadate mixtures in Mg(NO_3_)_2_, which indicates a certain degree of structural arrangement/conversion of Mg-vanadate phases during cycling and possible synergy among vanadate phases in the mixed oxide. More pronounced electrochemical activity of the MgV_2_O_6_ phase towards insertion/deinsertion of Mg^2+^ and Al^3+^ ions in the MgVO-LT than in the sample with the pure MgV_2_O_6_ phase could result from the smaller crystal dimensions (45 nm crystallite size of MgV_2_O_6_ in MgVO-LT, instead of 57 nm in MgVO-HT) and particle size, but also indicates that some kind of synergistic effect among different phases present in the MgVO-LT composite is possible during the electrochemical processes, which would make performance of the multiphase composite better than those of individual phases, similarly to the catalytic activity of Mg-V-O materials [74]. 

This may be caused by the presence of oxygen vacancies and grain boundaries. Generated oxygen vacancies in multiphase material can shorten the path for Mg^2+^ ions (acting as channels for their diffusion) and/or improve the electronic wiring by changing the electronic cloud (induced by free electrons generated around vacancies) [22]. Besides, the phase boundary may help diffusion of Mg^2+^ ions [22], thus introducing defects and/or active sites [22,75]).

### 3.5. Structural, Morphological, and Electrochemical Characterization of the MgVO/C Material with Improved Performances

Taking into account poor redox kinetics of MgVO-HT (or MgV_2_O_6_), caused by low electronic and ionic wiring, the further step was directed towards the charge storage improvement of this phase. With this aim, carbon was incorporated into the MgVO-HT by mixing the material with sucrose and annealing the mixture at 700 °C for 2 h under Ar atmosphere, thus obtaining the material marked as MgVO/C.

**Figure 7:**
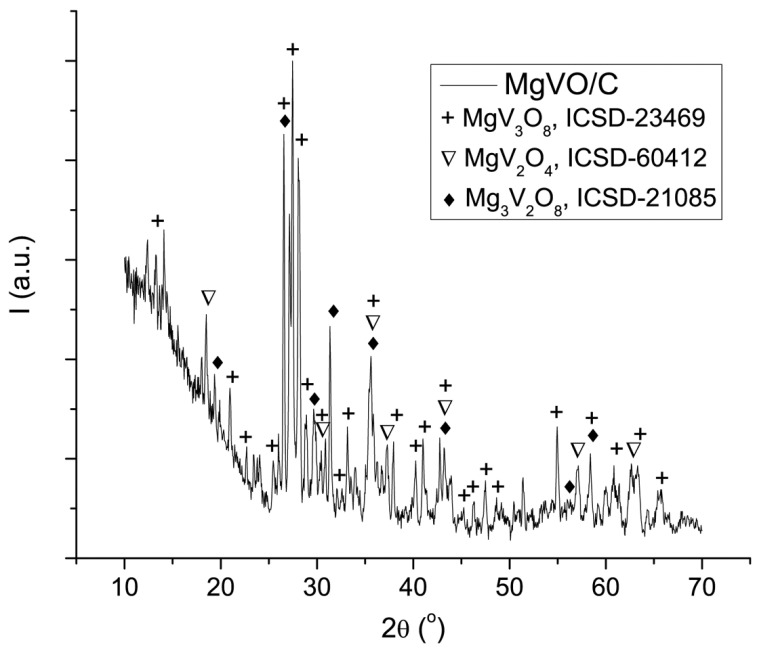
XRD patterns of the MgVO/C material.

**Figure 8:**
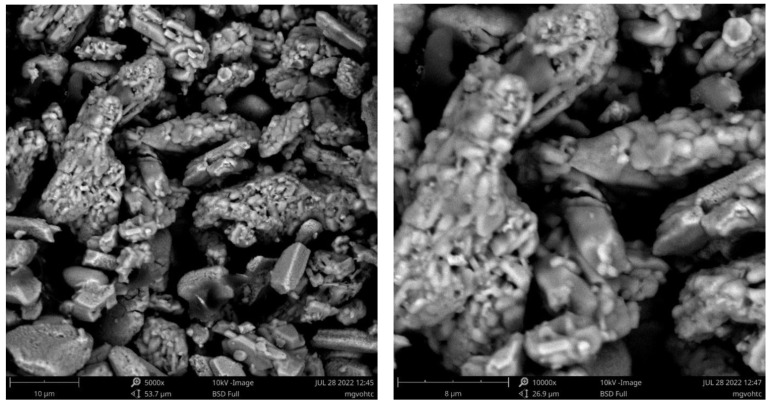
SEM micrographs of MgVO/C at different magnifications.

Structural analysis of the prepared composite powder revealed that the material changed the phase composition after thermal treatment with sucrose (Figure 7). The presence of MgV_3_O_8_, Mg_3_V_2_O_8_, and MgV_2_O_4_ crystalline phases in the sample, with weight fractions (without C) 54%, 35%, and 11%, respectively, is observed. The sucrose addition also changes the morphology of sample (Figure 8). Plate microagglomerates (~10 μm) were retained upon carbon formation, but their surface became more porous and rougher. As can been seen from Figure 8, MgVO/C agglomerates are composed from irregular nano- and microparticles, formed as a consequence of the carbon reduction treatment. This indicates not only improved electronic conductivity but also enhanced diffusion behavior. 

**Figure 9:**
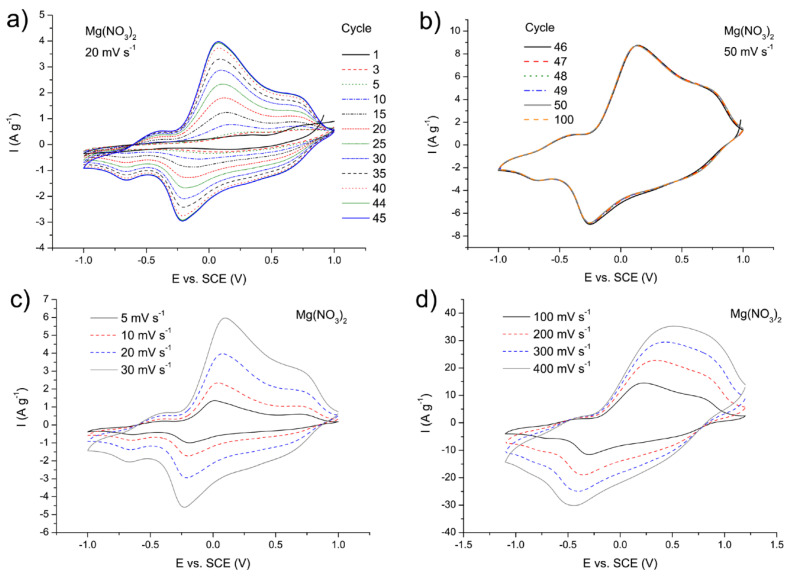
Cyclic voltammograms of the MgVO/C recorded in 3M Mg(NO_3_)_2_ solution: at 20 mV s^−1^ (**a**); at 50 mV s^−1^ after 45 successive cycles previously recorded at 20 mV s^−1^ using the same working electrode (**b**); at 5–30 mV s^−1^ (**c**); at 100–400 mV s^−1^ (**d**).

Electrochemical test of the MgVO/C in 3M Mg(NO_3_)_2_ solution performed by cyclic voltammetry measurements at 20 mV s^−1^ showed the presence of redox peaks (Figure 9a), which correspond to valence state transition of vanadium together with insertion/deinsertion of Mg^2+^ into/from the structure of the MgVO/C [18]. Increase in the current values including further activation of the material was observed with each subsequent measurement cycle, up to the 45th cycle, when the current became stabilized (Figure 9a). The maximal current after stabilization is around 40 times higher than that of the MgVO-HT, and twice as high as that of the MgVO-LT material (Figure 4a,e and Figure 9a). Cyclic voltammograms (5 cycles) recorded afterwards at 50 mV s^−1^ with the same working electrode confirmed the stability of the host material during Mg^2+^ insertion/deinsertion (Figure 9b). Gradual activation of the material (CVs at 20 mV s^−1^), similarly to the MgVO-LT, probably resulted from coinsertion of water molecules into the structure of MgVO/C, together with Mg^2+^ ions, due to which the high charge density of Mg^2+^ became buffered [8], allowing faster cation transfer. Similarly to the previously described materials MgVO-HT and MgVO-LT, redox peak positions of MgVO/C indicate that inserted Mg^2+^ ions occupied the sites in the structure near the center of square-planar oxygen atoms and around apical oxygen atoms [40]. Cyclic voltammograms recorded at lower and higher scanning rates revealed well-defined redox peaks up to the scanning rate of 100 mV s^−1^ (Figure 9c,d), suggesting the ability of the material to tolerate relatively high currents. However, at scanning rates higher than 100 mV s^−1^, individual peaks in both directions became merged, since the Mg^2+^ ions did not have enough time to completely extract from and insert into the material within the used potential range, causing irreversible behavior.

The MgV_3_O_8_ phase, present as the most abundant phase of the MgVO/C material, is significant for the electrochemical activity of the sample towards insertion/deinsertion of Mg^2+^ ions. This phase is characterized by the mixed valence (V^5+^/V^4+^), which is considered to optimize electron conductivity and give various redox sites for the electrochemical process [76]. Further, the MgV_2_O_4_ phase, although present in minor amounts, is supposed to have complex defect chemistry, including the presence of some V^5+^/V^4+^ beside the V^3+^ in the material [28], which additionally contributed to the electrochemical activity of the studied material. Thus, in spite of the fact that 35% of the MgVO/C sample is an inactive phase (Mg_3_V_2_O_8_, Figure S4b (Supplementary Materials)), the MgVO/C exhibited improved electrochemical activity towards insertion/deinsertion of Mg^2+^ ions compared with other simply prepared Mg-V-O materials studied in this work.

**Figure 10:**
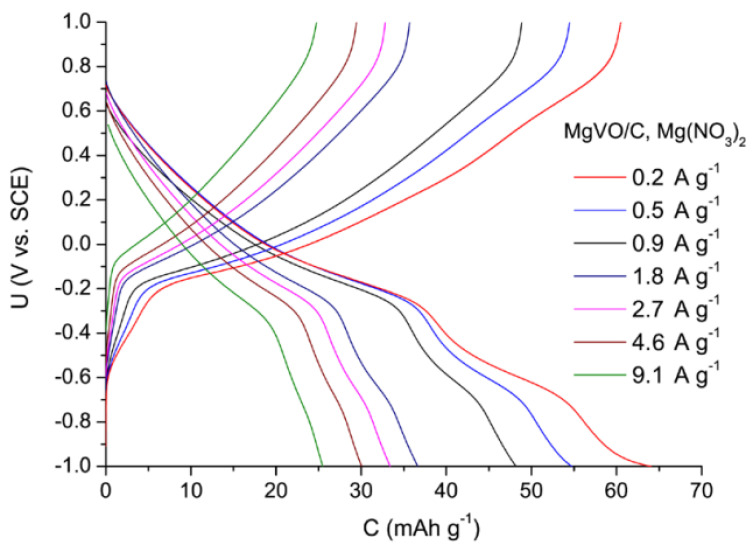
Chronopotentiometric curves of the MgVO/C in 3M Mg(NO_3_)_2_, recorded at different current values.

After stabilization, chronopotentiometric curves were recorded. The voltage-capacity profile exhibits two waves (Figure 10), which suggests the multistep Mg^2+^ ion insertion behavior [29]. Specific capacity of around 50 mAh g^−1^ was reached at 0.91 A g^−1^. Subsequent charge/discharge curves are very similar. Still, the capacity is inferior when compared with the values of some reported vanadium oxides [40,41,77,78,79] measured in Mg-containing aqueous electrolytes (Table S2).

#### 3.5.1. BVS Calculations for the Phases Present in the MgVO/C

**Figure 11:**
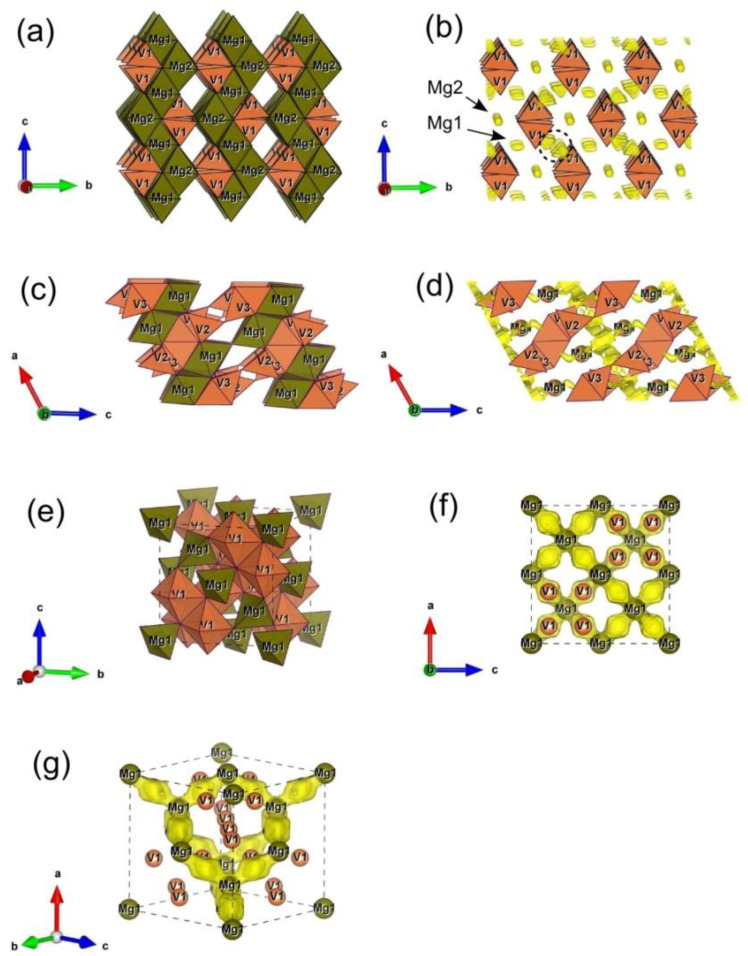
Models of migration pathways for Mg^2+^ ion obtained by BVS analysis of vanadate phase constituents of MgVO/C. Polyhedral representation of Mg_3_V_2_O_8_ (**a**) and BVS map within its structure (**b**); polyhedral representation of β-MgV_3_O_8_ (**c**) and BVS map within its structure (**d**); polyhedral representation of MgV_2_O_4_ (**e**) and BVS map within its structure viewed from different directions (**f**,**g**). BVS volumes are defined by ±0.1 v.u. mismatch from the valence +2 of Mg.

Further explanation of the electrochemical behavior of the MgVO/C was given from the point of view of possible Mg^2+^ diffusion pathways in the structures of interest, utilizing the bond valence method. In the presented study, crystallographic data of Mg_3_V_2_O_8_ (orthorhombic s.g. *Cmca* [80]), MgV_3_O_8_ (monoclinic s.g. *C*2/*m* [81]), and MgV_2_O_4_ (cubic s.g. *Fd*-3*m* [82]) phases have been used as input for BVS calculation and results are presented in Figure 11.

In magnesium orthovanadate, Mg_3_V_2_O_8_, Mg^2+^ occupies two types of octahedral sites, Mg1 and Mg2 crystallographic position, thus forming layers of edge-sharing Mg1O_6_ and Mg2O_6_ octahedra in ac-plane; the MgO_n_ layers are separated by V1O_4_ tetrahedrons, as depicted in Figure 11a. Although transport within the layer seems most probable, this is not the case, since there exists no continuous network of BVS isosurfaces in the structure of Mg_3_V_2_O_8_; there are only isolated “islands” of possible Mg^2+^ occurrence, including both Mg1 and Mg2 sites, and one additional interstitial position (encircled with dashed line in Figure 11b). This suggest that magnesium ions remain firmly trapped in their respective positions, making the structure more/less inactive for reversible magnesium extraction, as confirmed by electrochemical measurements (Figure S4b, Supplementary Materials).

In the second investigated structure, which is beta phase MgV_3_O_8_, magnesium occupies an octahedral position with symmetry: 1 (denoted as Mg1), which it randomly shares with one V^4+^ ion; edge- and corner-shared Mg1O_6_ octahedrons thus form layers normal to A-axis, which are pillared by V^5+^O_4_ tetrahedrons and V^5+^O_5_ hexahedrons, as presented in Figure 11c. The Mg^2+^ diffusion within this structure is achieved dominantly through a set of intersected tunnels in ab-plane, as could be seen in Figure 11d. Note that Mg1 position is located outside of the planar tunnel network, suggesting that partial occupation of this position by V^4+^ could not significantly affect migration of Mg^2+^ ions.

In the third structure of interest, which is spinel MgV_2_O_4_ (displayed in Figure 11e), Mg^2+^ and V^3+^ ion occupy one tetrahedral Mg1 and one octahedral V1 position, respectively. Mg1O_4_ shares only corners with neighboring V1O_6_ and has neither common corners nor edges with other Mg1O_4_. Still, for the applied BVS mismatch of ±0.1 v.u, Mg1 sites are interconnected through broad channels of BVS isosurfaces, thus providing an extensive tunnel network for 3D migration of Mg^2+^ ions (Figure 11f,g). According to the insights into possible Mg^2+^ diffusion pathways of the three structures of the MgVO/C material, provided by BVS calculations, the Mg^2+^ ion conduction capabilities of the investigated phases decrease in order: MgV_2_O_4_ > MgV_3_O_8_ > Mg_3_V_2_O_8_. 

Further, if we combine results obtained via BVS analysis of all aforementioned vanadate phases, they can be classified with respect to Mg^2+^ ionic diffusion capabilities as follows: (1) the MgV_2_O_4_ phases would fall into category of good Mg^2+^ ionic conductors; (2) MgV_3_O_8_ as a medium conductor; (3) Mg_2_VO_6_ as a poor conductor; while (4) Mg_3_V_2_O_8_ and α-Mg_2_V_2_O_7_ structures have no potential for reversible magnesium extraction. These findings could serve as a solid basis for future development of cathodes among different magnesium vanadate materials.

#### 3.5.2. Comparison of the MgVO/C Performance in the Mg^2+^, Al^3+^, and Li^+^ Electrolytes 

**Figure 12:**
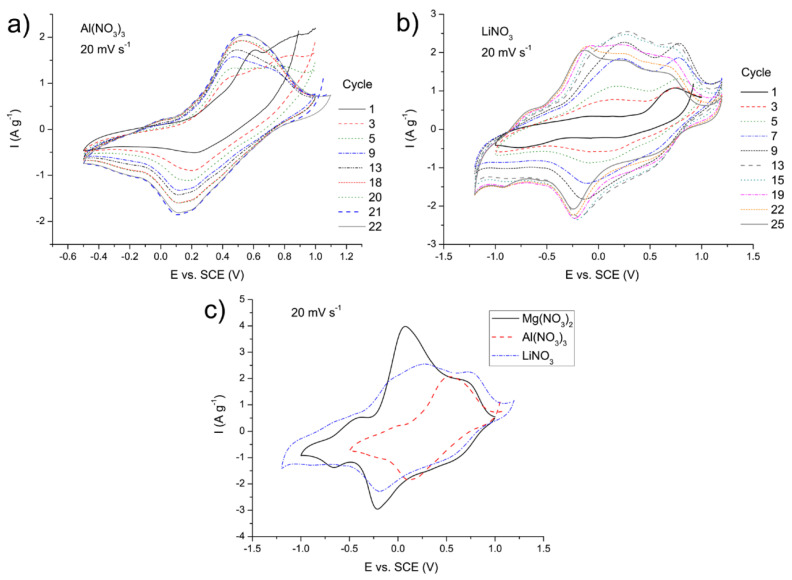
Cyclic voltammograms of the MgVO/C recorded at 20 mV s^−1^ in 1M Al(NO_3_)_3_ (**a**) and 6M LiNO_3_ (**b**), showing together the highest currents achieved in three different media (Mg, Al, and Li nitrate) (**c**).

For comparison purposes, the MgVO/C material was additionally electrochemically tested in 1M Al(NO_3_)_3_ and 6M LiNO_3_ by cyclic voltammetry measurements. Some similarities in the behavior of the CVs recorded in different media can be observed (Figure 9 and Figure 12). In the case of the Al(NO_3_)_3_ electrolyte, insertion/deinsertion of Al^3+^ ions occurred, while the values of current grew up to the 21st cycle, after which a decline in the current values took place in a manner similar to that of MgVO-LT (Figure 12a), resulting from partial damage of the electrode material, as explained in the previous part. However, the maximum current density achieved in the Al(NO_3_)_3_ is about twice lower than that achieved in the Mg(NO_3_)_2_ (Figure 12c). In the case of LiNO_3_ electrolyte and insertion/deinsertion of Li^+^ ions, growth of the current was observed up to the 13th cycle (Figure 12b), while the maximum current density was about 35% lower than that in Mg(NO_3_)_2_ (Figure 12c). Higher stability of the Mg-V-O materials in Mg^2+^ electrolyte than in the other ones could, to a large extent, result from the higher tendency of Mg^2+^ ions to occupy the sites around apical oxygen atoms than that of the Al^3+^ and Li^+^ ions, leading to redistribution of charge and reduced tension of crystal lattices [40]. Certain changes in the shape of the CVs in Al(NO_3_)_3_ and LiNO_3_ observed with the progress of cycling suggested that some of the inserted Al^3+^ and Li^+^ ions could not be completely extracted from the starting MgVO/C structure [83], which could also include an irreversible phase transition during the electrochemical cycling [46].

## 4. Conclusions

MgVO materials of various structures, synthesized by simple preparation route including precipitation followed by thermal annealing, were studied as potential cathode materials for rechargeable aqueous magnesium ion batteries. 

Redox behavior of the examined materials was shown to be complex and dependent on the structure (phase composition). Multiphase oxide systems (MgVO-LT and MgVO/C) exhibited better Mg^2+^ insertion/deinsertion performances than the single-phase ones (MgV_2_O_6_ and Mg_2_V_2_O_7_). It was suggested that some kind of synergistic effect among different phases of MgVO-LT is possible during the electrochemical processes, in spite of the slow Mg^2+^ ion diffusion in the MgV_2_O_6_ and Mg_2_V_2_O_7_ phases.

To improve redox activity of the MgV_2_O_6_ phase, carbon was integrated with the MgVO-HT material by sucrose-assisted thermal treatment, yielding MgVO/C. Carbon addition was shown to be an effective dual strategy for enhancing electrochemical performance of the studied MgVO materials. Role of carbon included improvement of electrical conductivity of the magnesium vanadium oxide sample as well as the phase transformation into phases with better Mg^2+^ diffusion properties than those of the MgV_2_O_6_ phase. Better electrochemical activity for insertion/deinsertion of metal ions and higher stability of MgVO/C was observed in the Mg^2+^ electrolyte compared with the Al^3+^ and Li^+^ ones, probably because of the greater tendency of Mg^2+^ ions to occupy the sites around apical oxygen atoms than that of the Al^3+^ and Li^+^, and resulting reduced tension of crystal lattices in the case of Mg^2+^ electrolyte. All this makes the magnesium vanadium oxide materials promising for further development with a view to their application in rechargeable aqueous magnesium ion batteries.

## Figures and Tables

**Table 1:** Assignation of the FTIR and Raman spectral bands of the MgVO-HT material.

Band Position	Assignation	Reference
FTIR spectra		
885 and 815 cm^−1^	MgV_2_O_6_-stretching asymmetric ν_as_(VO_4_) and ν_as_(VO_6_)	[58,63]
540 cm^−1^	MgV_2_O_6_-stretching symmetric ν_s_(VOV)	[58]
425 cm^−1^	MgV_2_O_6_-bending symmetric δ_s_(VO_4_) and δ_s_(VO_6_); and Mg-O vibrations	[58,63]
Raman spectra		
925 cm^−1^	MgV_2_O_6_-stretching ν(V^V^=O) and ν_s_(VO_4_) (symmetric)	[58]
840 cm^−1^	MgV_2_O_6_-stretching asymmetric ν_as_(VO_4_) and ν_as_(VO_6_)	[58]
733 cm^−1^	MgV_2_O_6_-stretching asymmetric ν_as_(VOV)	[58]
524 cm^−1^	MgV_2_O_6_-stretching symmetric ν_s_(VOV)	[58]
444 cm^−1^	MgV_2_O_6_-bending symmetric δ_s_(VO_4_) and δ_s_(VO_6_)	[58]
270–330 cm^−1^	MgV_2_O_6_-bending asymmetric δ_as_(VO_4_) and δ_as_(VO_6_)	[58,61]
205 cm^−1^; 175 cm^−1^; 150 cm^−1^	MgV_2_O_6_	[59,61]

**Table 2:** Assignation of the FTIR and Raman spectral bands of the MgVO-LT material.

Band Position	Assignation	Reference
FTIR spectra		
1020 cm^−1^	V_2_O_5_-stretching ν(V-O)	[58]
975 cm^−1^	Mg_2_V_2_O_7_-stretching ν(V^V^=O) and ν_s_(VO_4_) (symmetric)	[58]
815–920 cm^−1^	V_2_O_5_, MgV_2_O_6_, and Mg_2_V_2_O_7_-stretching asymmetric ν_as_(VO_4_) and ν_as_(VO_6_)	[58]
620–720 cm^−1^	V_2_O_5_ and Mg_2_V_2_O_7_-stretching asymmetric ν_as_(VOV)	[58,60,61]
540 cm^−1^	MgV_2_O_6_-stretching symmetric ν_s_(VOV)	[58]
420–450 cm^−1^	V_2_O_5_, MgV_2_O_6_, and Mg_2_V_2_O_7_-bending symmetric δ_s_(VO_4_) and δ_s_(VO_6_)	[58]
Raman spectra		
996 cm^−1^	V_2_O_5_-stretching ν(V^V^=O) and ν_s_(VO_4_) (symmetric)	[58]
922 cm^−1^	MgV_2_O_6_-stretching ν(V^V^=O) and ν_s_(VO_4_) (symmetric)	[58,59]
840 cm^−1^	V_2_O_5_ and MgV_2_O_6_-stretching asymmetric ν_as_(VO_4_) and ν_as_(VO_6_); Mg_2_V_2_O_7_- stretching asymmetric ν_as_(VOV)	[58]
730 cm^−1^	V_2_O_5_ and MgV_2_O_6_-stretching asymmetric ν_as_(VOV)	[58,59]
523 cm^−1^	MgV_2_O_6_ and Mg_2_V_2_O_7_-stretching symmetric ν_s_(VOV)	[58,59]
440 cm^−1^	MgV_2_O_6_ and Mg_2_V_2_O_7_-bending symmetric δ_s_(VO_4_) and δ_s_(VO_6_)	[58]
412 cm^−1^	V_2_O_5_-bending symmetric δ_s_(VO_4_) and δ_s_(VO_6_)	[58,59]
335 cm^−1^	V_2_O_5_ and Mg_2_V_2_O_7_-bending asymmetric δ_as_(VO_4_) and δ_as_(VO_6_)	[58]
310 cm^−1^	MgV_2_O_6_ and Mg_2_V_2_O_7_-bending asymmetric δ_as_(VO_4_) and δ_as_(VO_6_)	[58]
285 cm^−1^	Mg_2_V_2_O_7_-bending asymmetric δ_as_(VO_4_) and δ_as_(VO_6_)	[58]
272 cm^−1^	MgV_2_O_6_-bending asymmetric δ_as_(VO_4_) and δ_as_(VO_6_)	[58]
205 cm^−1^; 175 cm^−1^; 150 cm^−1^	MgV_2_O_6_	[59,61]

## Data Availability

Not applicable.

## Supplementary Materials

The following supporting information can be downloaded at: https://www.mdpi.com/article/10.3390/nano12162767/s1, Figure S1: SEM micrographs of MgVO-LT at different positions and magnifications; Figure S2: Cyclic voltammograms of MgVO-HT in 1M Al(NO_3)3_, at 20 mV s^–1^; Figure S3: Raman spectra of the MgVO-LT electrode material acquired at different spots, before and after electrochemical treatments in Mg(NO_3_)_2_ and Al(NO_3_)_3_; Figure S4: Cyclic voltammograms of Mg_2_V_2_O_7_ (a) and Mg_3_V_2_O_8_ (b) materials in 3 M Mg(NO_3_)_2_, at 20 mV s^–1^; Table S1: Assignation of the FTIR and Raman spectral bands of the MgVO precursor material; Table S2: Comparative electrochemical properties of VO-based oxides in Mg-containing aqueous solutions.
