# Supplementary material for: Simply Prepared Magnesium Vanadium Oxides as Cathode Materials for Rechargeable Aqueous Magnesium Ion Batteries
Source: Nanomaterials (Basel). 2022 Aug 12;12(16):2767. doi: 10.3390/nano12162767 (PMC9412870; doi:10.3390/nano12162767)
Supplement: Supplementary file 1 [file nanomaterials-12-02767-s001.zip › nanomaterials-1848656-supplementary.pdf]

Supplementary data

Simply Prepared Magnesium Vanadium Oxides as Cathode Materials for Rechargeable Aqueous Magnesium Ion Batteries

Milica M. Vasić [1], Miloš Milović [2], Danica Bajuk-Bogdanović [1], Tamara Petrović [1] and Milica J. Vujković [1,*]

[1] Faculty of Physical Chemistry, University of Belgrade, Studentski trg 12-16, 11000 Belgrade, Serbia

[2] Institute of Technical Sciences of SASA, Knez Mihajlova 35/IV, 11000 Belgrade, Serbia

* Correspondence: milica.vujkovic@ffh.bg.ac.rs

Table S1. Assignation of the FTIR and Raman spectral bands of the MgVO precursor material.

**Band position**	**Assignation**	**reference**
FTIR spectra		
1417cm^{-1}	NH_4VO_3- stretching $\nu(NH_4^+)$	[62]
930 cm^{-1}	NH_4VO_3- stretching symmetric $\nu_s(VO_2)$	[62]
896 cm^{-1}	NH_4VO_3- stretching asymmetric $\nu_{as}(VO_2)$	[62]
850 cm^{-1}	NH_4VO_3	[62]
685 cm^{-1}	NH_4VO_3- stretching asymmetric $\nu_{as}(VOV)$	[62]
500 cm^{-1}	NH_4VO_3- stretching symmetric $\nu_s(VOV)$	[62]
	Raman spectra	
928cm^{-1}	NH_4VO_3- stretching symmetric $\nu_s(VO_2)$	[62]
897cm^{-1}	NH_4VO_3- stretching asymmetric $\nu_{as}(VO_2)$	[62]
646 cm^{-1}	NH_4VO_3- stretching asymmetric $\nu_{as}(VOV)$	[62]
496 cm^{-1}	NH_4VO_3- stretching symmetric $\nu_s(VOV)$	[62]
383 cm^{-1}; 350cm^{-1}	NH_4VO_3	[62]
322 cm^{-1}	NH_4VO_3- bending $\delta(VO_2)$	[62]
259 cm^{-1}; 213 cm^{-1}	NH_4VO_3	[62]

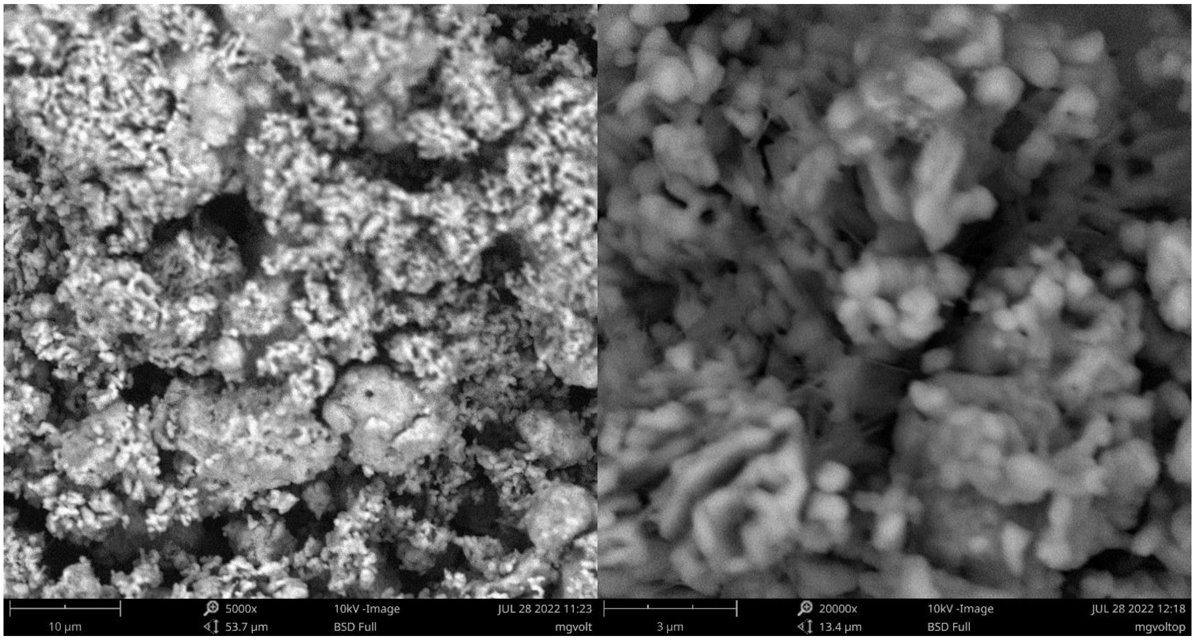

Figure S1. SEM micrographs of MgVO-LT at different positions and magnifications.

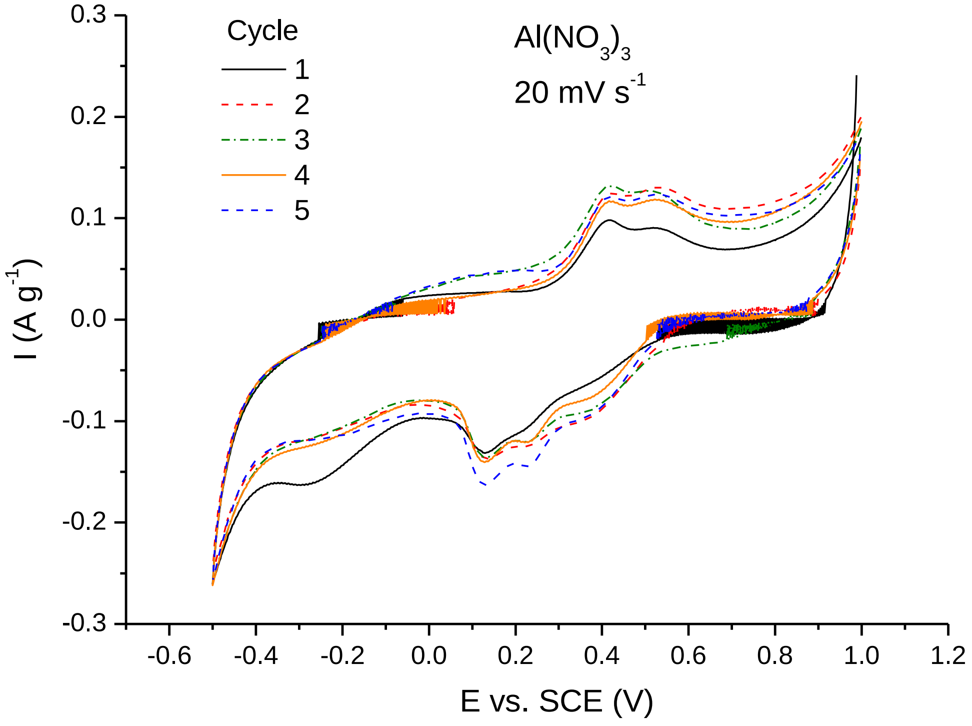

Figure S2. Cyclic voltammograms of MgVO-HT in 1M $Al(NO_3)_3$, at 20 mV s^{-1}.

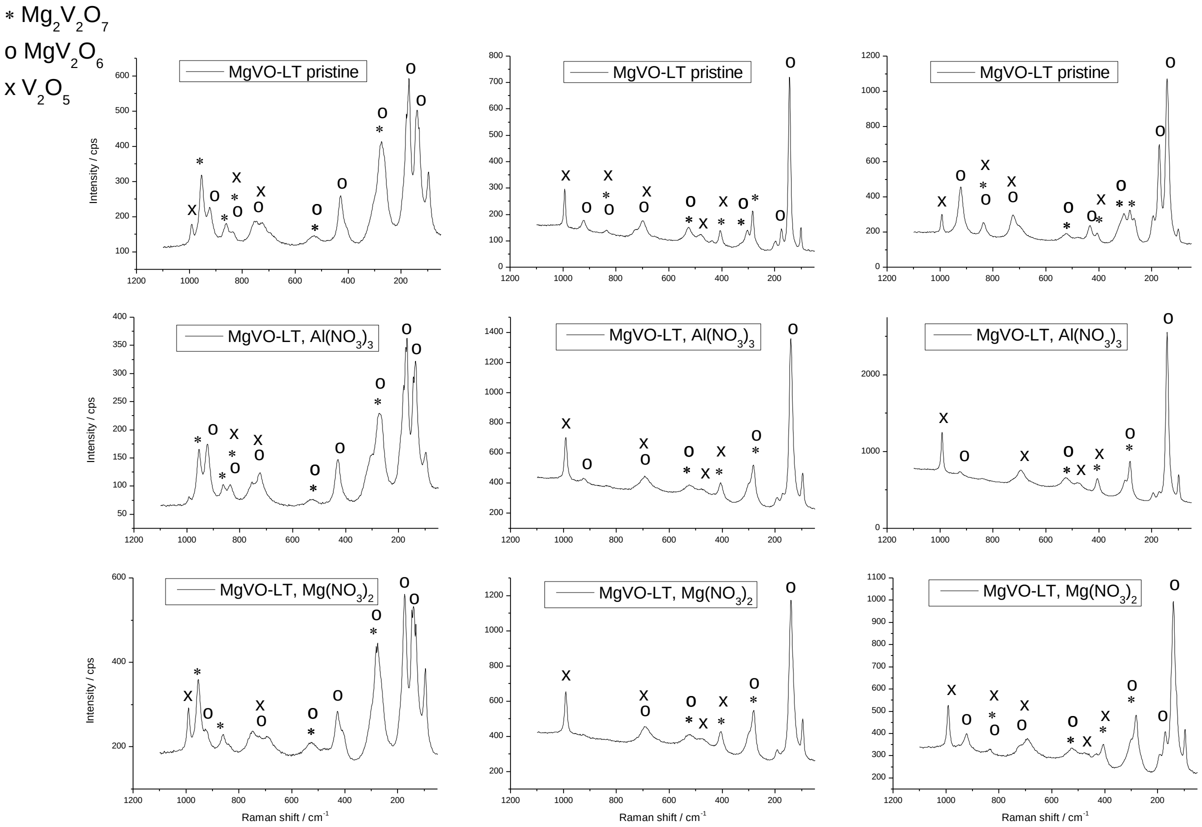

Figure S3. Raman spectra of the MgVO-LT electrode material acquired at different spots, before and after electrochemical treatments in $Mg(NO_3)_2$ and $Al(NO_3)_3$.

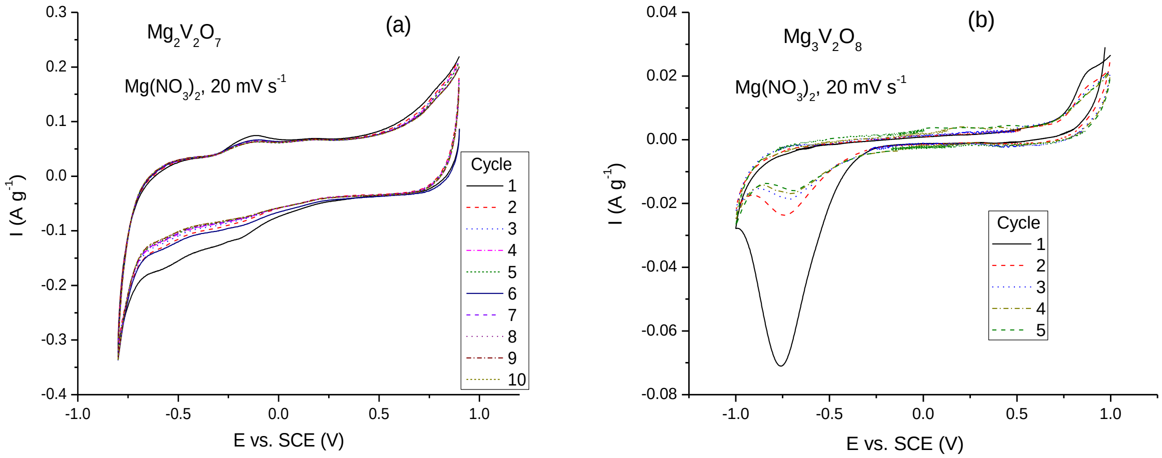

Figure S4. Cyclic voltammograms of $Mg_2V_2O_7$ (a) and $Mg_3V_2O_8$ (b) materials in 3 M $Mg(NO_3)_2$, at 20 mV s^{-1}.

Table S2. Comparative electrochemical properties of VO-based oxides in Mg-containing aqueous solutions.

Material	Electrolyte	Capacity / mAh g^{-1}	Current / A g^{-1}	Ref.
MgV_3O_8,/$Mg_3V_2O_8$/MgV2O4 cathode (MgVO/C)	3 M $Mg(NO_3)_2$	65<br>48<br>30	0.2<br>0.9<br>4.6	This work
V_2O_5/MgV_2O_6/$Mg_2V_2O_7$ cathode (MgVO-LT)	3 M $Mg(NO_3)_2$	35<br>20<br>14	0.1<br>0.5<br>5	This work
V_2O_5 cathode<br>AACVD* synt. at 500°C	0.075 M $MgCl_2$	427<br>225<br>170	5.9<br>3.9<br>2.4	[77]
V_2O_5,<br>AACVD* synt. at 600°C<br>(cathode)	0.075 M $MgCl_2$	300<br>270<br>230	15<br>4.9<br>3.9	[78]
V_2O_5 xerogel/graphite cathode	3 M $Mg(NO_3)_2$	100<br>53<br>44	1<br>3<br>5	[40]
NaV_6O_{15}/NaV_6O_{16}	3 M $Mg(NO_3)_2$	109	20<br>mVs^{-1}	[41]
$FeVO_4$ anode	1 M $MgSO_4$	140<br>100<br>51	0.05<br>0.1<br>0.5	[79]
$FeVO_4$/C anode	1 M $MgSO_4$	184<br>135<br>88 mAh g^{-1}	0.05<br>0.1<br>0.5	[79]

*AACVD – aerosol assisted chemical vapor deposition
